# A Portable Triboelectric Nanogenerator for Real-Time Respiration Monitoring

**DOI:** 10.1186/s11671-019-3187-4

**Published:** 2019-11-28

**Authors:** Zhicheng Zhang, Jiwei Zhang, He Zhang, Huagang Wang, Zhiwei Hu, Weipeng Xuan, Shurong Dong, Jikui Luo

**Affiliations:** 1 College of Civil Engineering & Architecture, Zhejiang University, 866 Yuhangtang Road, Hangzhou, 310058 China; 2 Ministry of Education Key Lab. of RF Circuits and Systems, College of Electronics & Information Hangzhou Dianzi University, Hangzhou, 310018 China; 3 College of Information Science & Electronic Engineering, Zhejiang University, 38 Zheda Road, Hangzhou, 310027 China; 4 Institute of Renewable Energy & Environmental Technology, Bolton University, Deane Road, Bolton, BL3 5AB UK

**Keywords:** Waist-wearable respiration sensor, Triboelectric nanogenerator, Wireless transmission

## Abstract

As a reliable indicator of human physiological health, respiratory rate has been utilized in more and more cases for prediction and diagnosis of potential respiratory diseases and the respiratory dysfunction caused by cystic fibrosis. However, compared with smart mobile electronics, traditional clinical respiration monitoring systems is not convenient to work as a household wearable device for real-time respiration monitoring in daily life due to its cumbersome structure, complex operability, and reliance on external power sources. Thus, we propose a wearable wireless respiration sensor based on lateral sliding mode triboelectric nanogenerator (TENG) to monitor respiratory rates by sensing the variation of the abdominal circumference. In this paper, we validate the possibility of the device as a respiration monitoring sensor via an established theoretical model and investigate the output performance of the sensor via a series of mechanical tests. Furtherly, the applications of the respiration sensor in different individuals, different breathing rhythms, different active states, and wireless transmission have been verified by a lot of volunteer tests. All the results demonstrate the potential of the proposed wearable sensor as a new alternative for detecting and monitoring real-time respiratory rates with general applicability and sensitivity.

## Introduction

Accompanied with the global climate deterioration, increasing serious air pollution and the aggravation tendency of aged population, the human health, especially the health of the respiratory system, is exposed to more and more threats [1–3]. Meanwhile, the monitoring for human’s physical health becomes the focus of attention for preventing latent diseases [4–7]. Respiratory rate, as one of the most important and reliable indicators directly reflecting human physiological health, may provide key information for the prediction and diagnosis of potential respiratory diseases like obstructive sleep apnea syndrome (OSAS) and the respiratory dysfunction caused by cystic fibrosis [8–11]. There has been various traditional medical equipment utilized for monitoring respiration status, and extraordinary efforts have also been committed to develop technologies for innovative respiration monitoring. Despite the great clinical applicability and monitoring accuracy, the cumbersome structure, complex operability, reliance on external power sources, and bad portability restrict their further development as smart mobile medical electronics. In recent years, the advances in mobile network and low-power electronics have driven the intelligent mobile medical devices at a tremendous pace and have evoked increasing interest in household healthcare and flexible wearable electronics [6, 12–18]. Therefore, the battery-free wearable healthcare sensors with great potential for respiration monitoring, in a smart way, are ubiquitously demanded.

Compared to some relatively mature bioenergy scavenging technologies like electromagnetic [19, 20] and piezoelectric [21–25], triboelectric nanogenerators (TENGs) [26–30], with the merits of light weight, high density of energy, and high sensing sensitivity, possess better potential in applications as bioenergy harvesters, wearable electronics, and self-powered health monitoring devices. Furtherly, the TENG-based energy harvesters are more capable in scavenging bioenergy in working environment with the bandwidth of frequency below 10 Hz like human breath [31, 32], and the materials used for TENGs are lead free which are safe to be used for healthcare sensors. Therefore, TENG is of no doubt one of the best choices for wearable and self-powered breathing monitoring devices. To meet the increasing demands for wearable and self-powered health monitoring technology, many novel TENG-based sensors have been developed to monitor human physiological status. Lin et al. proposed a self-powered wireless body sensor network (BSN) system for heart rate monitoring *via* integration of a downy structure-based TENG (D-TENG), a power management circuit, a TENG-based heart rate sensor, a signal processing unit, and Bluetooth module for wireless data transmission in 2018 [13]. P. Maharjan et al. designed a novel curve-shaped wearable hybridized electromagnetic-TENG (WHEM-TENG) in 2018, working as an electronic wrist watch powered by biomechanical energy harvested from a swing arm, which was also demonstrated to power for a pulse signal and heart rate monitoring [17]. Chen et al. reported a flexible hybrid nanogenerator of piezoelectric and triboelectric properties in 2017 that can be conformally attached on soft surfaces like human skin to harvest diversity touch energies based on electrospun nanofiber mat and monitor the real-time physiological signals such as respiratory information and radial artery pulse [33]. Cu et al. reported a pulse sensor based on a single-electrode TENG with high flexibility and comfortability to human skin in 2018, with which a typical human pulse waveform that represents the radial artery pressure wave can be successfully obtained [34]. The abovementioned works have greatly propelled the development of TENG-based wearable and self-powered intelligent devices in human physical monitoring.

The variation of abdominal circumference is a natural physical behavior of human during breathing process so that capturing information from abdominal deformations is a sensing approach and has no negative effect on normal activities of human beings, which may also be a possible energy source by scavenging biokinetic energy. In this paper, we propose an integrated waist-wearable wireless respiration sensor based on sliding mode TENG, with the merits of portability, mobility, and intelligence, simultaneously. It may be applied in different daily activities for continuous real-time respiration monitoring and OSAS detecting with neither adverse effect on the normal function of the device nor adverse influence on daily activities of the user. A smart belt is built with the TENG sensor to sense the variation of the user’s abdominal circumference during breathing and transfer the periodic variation to the reciprocating oscillation of the tribo-pair of the TENG, so that the electric signals containing respiration information can be output by the TENG. The whole sensing process needs no external power source. The device is also equipped with a wireless transmission chip powered by an external source to realize respiration signal transmission. The information for the breath status will be finally displayed on a mobile phone. Here, we report the research work on the TENG-based respiration sensor to show its excellent potential as a possible intelligent wearable and self-powered device for real-time respiration monitoring.

## Methods

### Architecture of the Respiration Sensor

**Fig. 1:**
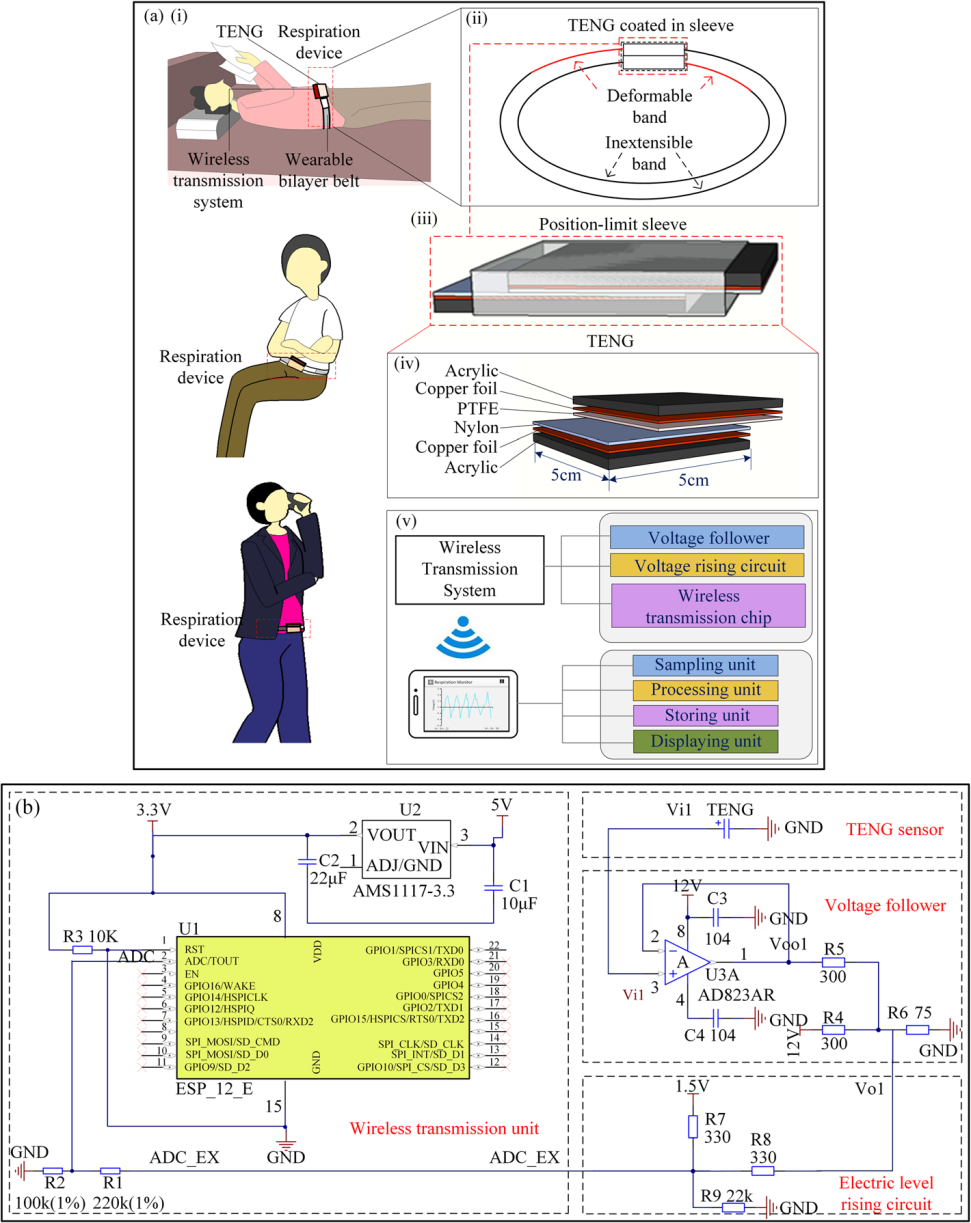
Fabrication of the waist-wearable respiration sensor and wireless transmission system. **a** Schematic design of the wireless respiration sensor. (i) Schematic for wearing, (ii) structural sketch of the wearable device, (iii) expanded view of the TENG and (iv) material illustration of the TENG, and (v) Functional modules contained in the wireless transmission system. **b** The circuit diagram of the wireless transmission system

Figure 1a shows the schematic structure of the respiration sensor based on sliding mode TENG. The waist-wearable respiration sensor is designed to detect the user’s real-time respiratory status in daily life, as shown in Fig. 1a (i). This kind of monitoring strategy will not disturb daily activities of the wearer, such as walking, sleeping, cooking, office work, etc. The device is composed of a wearable bilayer belt, a sliding mode TENG sensor built in the belt, and a wireless transmission system. Each layer of the bilayer belt, as shown in Fig. 1a (ii), includes an inextensible part noted by the black line and a deformable part by the red line. The TENG sensor is built in the wearable bilayer belt with the detailed structure shown in Fig. 1a (iii-iv). A polytetrafluoroethylene (PTFE) film with a thickness of 100 μm and a nylon film with a thickness of 30 μm are employed as the negative and positive tribo-materials, respectively. Two copper foils each with a thickness of 50 μm are attached on the outer surfaces of the tribo-layers as the conductive electrodes. Two acrylic sheets are utilized as the supports to keep the dielectric materials flat. The planer size of the TENG device is 5 × 5 cm^2^. The TENG device is coated in a plastic sleeve to ensure the contact between the tribo-pair during the respiration monitoring process.

The structure of the device is designed with a series of obvious merits. First of all, the deformable parts of the belt are utilized here to accommodate the expansion of the abdomen during respiration and offer the restoring force in the contracting procedure of the abdomen during inhalation process, so that the real-time detection with continuous signal will be realized via the smart belt with no uncomfortable feelings and negative influence on normal activities of the user. Secondly, the inextensible parts of the belt are used to restrict the deformation of the belt to make sure part of the abdominal circumference variation is used to drive the sliding behavior of the tribo-pair. Also, the simple structure and the commercial materials adopted in the device make it low cost and easy to fabricate, which may facilitate its marketable promotion prospect.

Furthermore, a set of hardware and software modules are applied to form a wireless transmission system for signal transmission, and the information of the real-time respiration is assumed to be displayed on a mobile phone (Fig. 1a (v)). As shown in Fig. 1b, the hardware module, consisting of a voltage follower, a voltage rising circuit, and a wireless transmission chip, are integrated into a circuit board. It is noticed that the TENG outputs high voltage but relatively low current, resulting in a high output impedance and affecting its applicability in the wireless transmission system. In this regard, the voltage follower is integrated in the circuit to lower the output impedance of the TENG so that it can roughly match that of the wireless transmission unit. Also, as a concern for practical applicability, the electric output of the TENG is characterized as alternating current, of which the negative signal values cannot be used as the input signal for the Analog Digital Converter (ADC). Therefore, the electrical level-rising circuit is used to elevate the whole signal curve of the output voltage of the TENG to positive level for the ADC to acquire the whole signals. The wireless transmission chip consists of an ADC, a microprocessor, an antenna, and a battery to provide power for the unit. The software module includes signal sampling, signal processing, signal storing, and signal displaying units. Through the signal sampling and processing units, the signals transmitted to the mobile phone are converted back to the oscillation with positive and negative components, but the signal waveforms and amplitudes are not converted back proportionally to the original values of the TENG output; thus, it is only indicative of respiration rates. And through the signal displaying and signal storing units, the transmitted signals of the real-time respiration rates are systematically stored and displayed on a mobile phone.

### Sensing Principle and Working Mechanism

Human breathing is usually categorized into thoracic and abdominal breathing, and most of us use the first type in our daily life. During the thoracic breathing process, the abdomen cavity periodically expands and contracts as the exhalation and the inhalation processes occur, respectively, which may induce stretching and contraction of the wearable belt attached around the waist. Meanwhile, the tribo-pair is forced to slide outward and inward via the deformation of the abdomen circumference. During the reciprocating sliding process, the respiration status will be obtained via the smart belt with the TENG device.

**Fig. 2:**
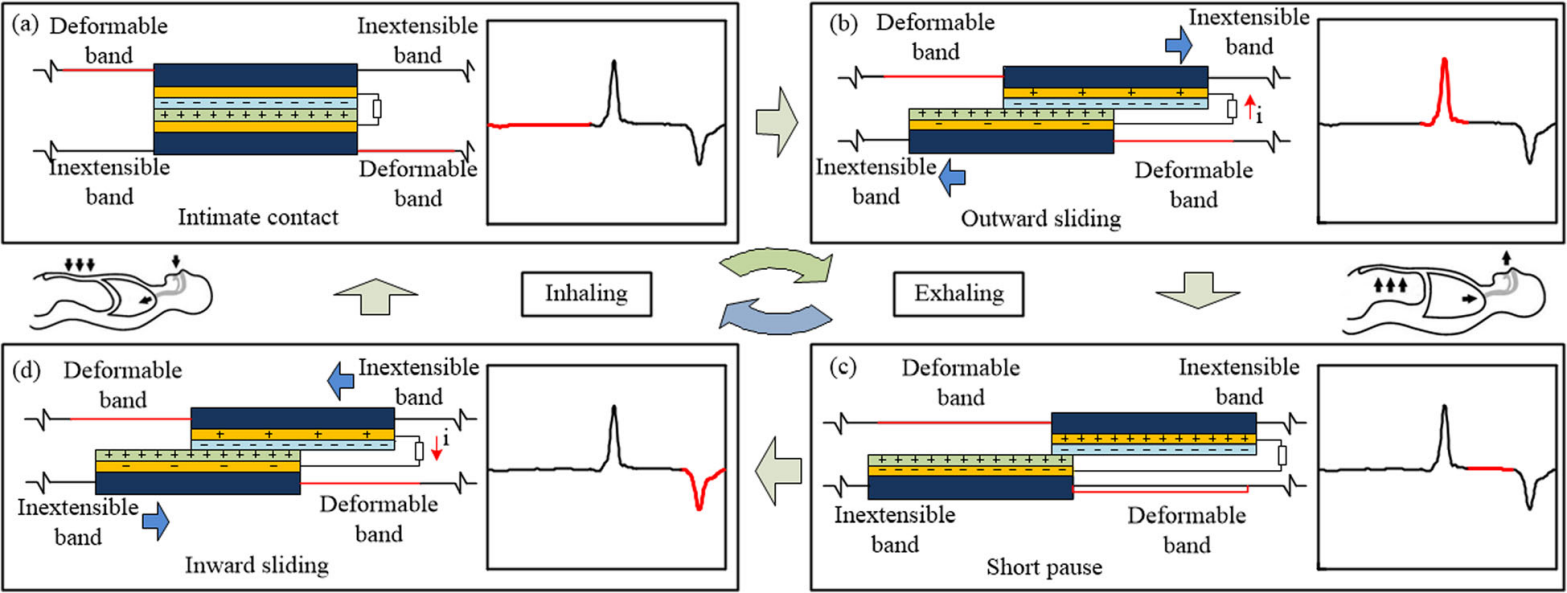
Working mechanism diagram of the respiration sensor and its four working processes. **a** “Intimate contact” process: user inhales and the surfaces of the tribo-pair fully overlap. **b** “Outward sliding” process: user exhales and the tribo-pair slides outward. **c** “Short pause” process: user exhales and the tribo-pair slides outward to the maximum extent. **d** “Inward sliding” process: user inhales and the tribo-pair slides inward

Figure 2 shows the working mechanism of the respiration sensor based on sliding mode TENG. The variation of the abdominal cavity circumference may facilitate the relative sliding of the tribo-pair via the wearable bilayer belt, inducing an alternating current passing through the external circuit, which will be captured and treated as the signal in the respiration monitoring. In each working cycle, there will be four processes: an initial intimate contact, an outward sliding, a short pause, and an inward sliding. As the initial state shown in Fig. 2a, the surfaces of the tribo-pair fully overlap and intimately contact with each other, and the surface of the nylon film and the PTFE film are positively and negatively charged, respectively, due to the triboelectric effect and electrostatic induction. In this stage, there is no sliding behavior between the tribo-pair, of which the surface charges are in static balance with no charge transferring in the circuit. When exhalation starts and tribo-pair begins to slide outward with the expansion of abdominal cavity (Fig. 2b), the separation of the triboelectric charge will induce a potential difference between the tribo-pair. Hence, the free electrons will be transferred from one electrode to another via the external circuit and a pulse of the output voltage with positive amplitude will be generated. Once the tribo-pair reaches the maximal sliding distance during the exhalation process (Fig. 2c), the transferred charges will reach its peak value and there will be no more current going through the circuit. Then comes the inhalation process (Fig. 2d), in which the tribo-pair begins to slide inward with the contraction of abdominal cavity. The redundant transferred charges on electrodes will flow back for new electrostatic balance and a pulse of the output voltage with negative amplitude will be generated. There will be no charge transferred as the charged surfaces of the tribo-pair get fully overlapped, and the TENG device will go back to the state of intimate contact as shown in Fig. 2a. In this way, with the occurrence of the repeated outward and inward oscillations between the tribo-pair, the electrons are driven forth and back in the circuit between the two electrodes, creating an alternating current output.

### Measurement System

The electrical output performances of the respiration sensor were recorded by a Keysight B2983A system electrometer.

## Results and Discussion

For clinical applications, respiratory rates may provide vital information for early warning and prompt diagnosis of the respiratory diseases like OSAS. The waist-wearable wireless respiration sensor is proposed in this paper to offer an alternative strategy for monitoring real-time respiration by sensing the variation of the abdominal circumference in the breathing process and displaying the wireless signal on a mobile phone. The configuration of the device contains a wearable bilayer belt, a sliding mode TENG sensor built in the belt and a wireless transmission system. And the applicability, portability, and accuracy of the device have been validated through theoretical analyses, mechanical tests and real-time tests by volunteers.

### Theoretical Prediction

**Fig. 3:**
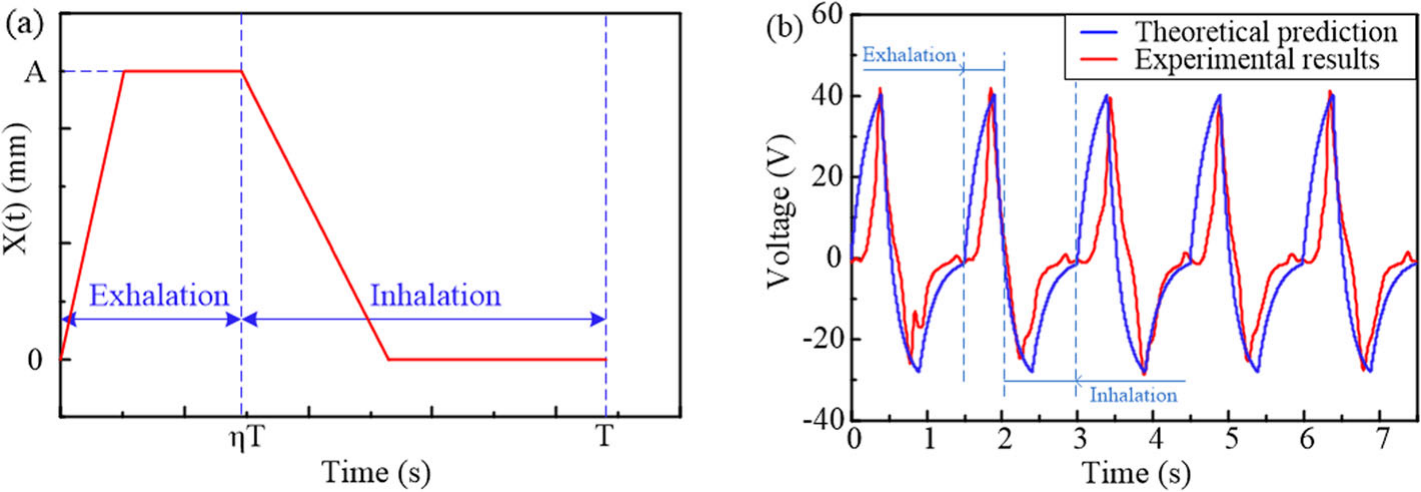
Correlation between the physical motion while breathing and the output voltage of the TENG sensor. **a** The assumed trapezoidal form of displacement for the theoretical prediction. **b** Comparison of the theoretical prediction and the experimental results

Firstly an analytical model is established to predict the output performance of the TENG and validate the possibility of the device as a respiration monitoring sensor. A real-time test is carried out to examine the accuracy of the analytical model. Furtherly, the correlation between the electrical signals of the sensor and input mechanical excitation is established and investigated by the theoretical model, which provides a better understanding of the working mechanism of the sensor. For those purposes, a theoretical function is proposed to simulate the breathing processes, which involves exhalation and inhalation stages. At the exhalation stage, the abdomen cavity expands and the tribo-pair slides outwards so that the displacement *x(t)* of the tribo-pair increases gradually from zero to *A*. Then the tribo-pair remains the maximum displacement *A* until the inhalation process. At the inhalation stage, the abdomen cavity contracts and the tribo-pair start to slide inwards, so that the displacement *x(t)* decreases gradually from *A* to zero. Afterwards, the tribo-pair remains the displacement of zero until the next breathing cycle. According to the variation regulation of the *x(t)* in time domain, the excitation for the device is assumed to be a trapezoidal wave (Fig. 3a), which is expressed as:

1 $$ x(t)=\left\{\begin{array}{c}{v}_1t\\ {}A\\ {}A-{v}_2t\\ {}0\end{array}\kern0.75em \begin{array}{c}0<t\le A/{v}_1\\ {}A/{v}_1<t\le \eta T\\ {}\eta T<t\le \eta T+A/{v}_2\\ {}\eta T+A/{v}_2<t\le T\end{array}\right. $$

where *T* is the period, *η* is the ratio of exhalation time to the whole period *T*, *v*_1_ and *v*_2_ are the speeds of sliding outwards and inwards, respectively. Furtherly, the output voltage *V*(*t*) is calculated according to the theory of the sliding mode TENG as follows [35, 36]:

2 $$ {\displaystyle \begin{array}{l}V(t)=\frac{\sigma {d}_0}{\varepsilon_0}\left[\frac{l}{l-x(t)}\exp \left(-\frac{d_0}{\varepsilon_0 RS}{\int}_0^t\frac{l}{l-x(t)}d{t}^{\prime}\right)\right.\\ {}\kern1.5em \left.+\frac{d_0}{\varepsilon_0 RS}\frac{l}{l-x(t)}{\int}_0^t\exp \left(\frac{d_0}{\varepsilon_0 RS}{\int}_t^{t^{\prime }}\frac{l}{l-x\left(\delta \right)} d\delta \right)d{t}^{\prime }-1\right]\end{array}} $$

where *d*_0_ = *d*_1_/*ε*_*r*1_ + *d*_2_/*ε*_*r*2_ is the effective thickness with *d*_1_ (*d*_2_) and *ε*_*r*1_ (*ε*_*r*2_) denoted the thickness and relative dielectric constant of the dielectric layer respectively, *ε*_0_ the dielectric constant of vacuum, σ the surface charge density, *R* the load resistance, and *S* the area of the dielectric plate.

A device is utilized as a case to validate the electromechanical model theoretically, with the parameters of the physical properties and loading process depicted in Tab. 1. The time history of the calculated output voltage is shown by the blue line in Fig. 3b, while the measured voltage signals, by the red line. Excellent agreement is observed between the theoretical prediction and the measured signals, suggesting that the analytical model is accurate for predicting the electric outputs of the device in the process of breathing. Furtherly, the voltage pulses of the predicted respiration signals show consistency with the inhalation and exhalation processes. The signals rise and fall, behaving with positive and negative signals with the occurrence of the exhalation and inhalation processes, respectively. And it may also be utilized for optimal design of the TENG-based respiration sensor in structural parameters to enhance the performance and sensitivity.

**Table 1:** Device parameters used in the experiment and simulation

Parameter	Value
Dielectric tribo-pair	PTFE-nylon
Thickness of nylon plate *d*_*1*_ (μm)	30
Relative permittivity of nylon *ε*_*r1*_	2.55
Thickness of PTFE plate *d*_*2*_ (μm)	100
Relative permittivity of PTFE *ε*_*r2*_	3.8
Permittivity of vacuum *ε*_*0*_ (pF/m)	8.854
Load resistance *R* (MΩ)	500
Area of the dielectrics *S* (cm^2^)	25
Surface charge density *σ* (μCm^−2^)	86
Maximum separate distance *A* (mm)	8
Cycle length of the excitation pattern *T* (s)	1.4952
Speed of sliding outwards / inwards *v*_1_ / *v*_2_ (m/s)	0.02/0.016
Ratio of the exhalation time to the whole breathing cycle *η*	0.267

### Output Characteristics

**Fig. 4:**
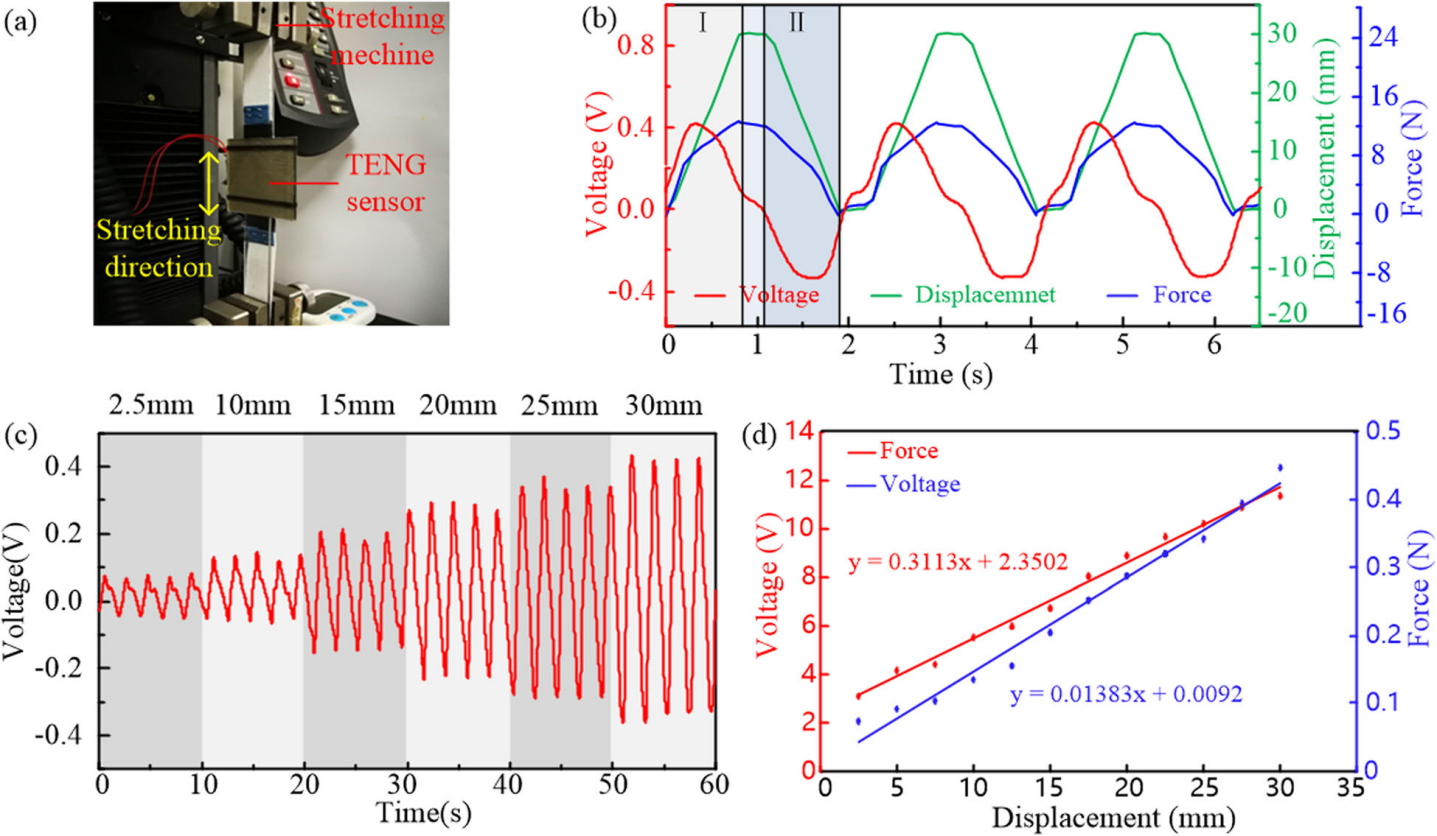
Mechanical tests on the TENG based respiration sensor. **a** Photograph of the TENG sensor fixed to the stretching machine. **b** The output voltage signals of the sensor under an excitation in trapezoidal form and the corresponding time histories of the sliding displacement and force. **c** The time histories of output voltage of the sensor with different displacement amplitudes. **d** The peak values of the output voltage and the traction force as functions of the maximum sliding displacement

A mechanical test has been carried out to investigate the influence of the sliding displacement of the tribo-pair on the output voltage signal of the device. As shown in Fig. 4a, the two ends of the tribo-pair were fixed on the stretching machine and the tribo-pair was forced into a periodic reciprocating sliding oscillation via the stretching machine to simulate the motion of the tribo-pair in the respiration process. Meanwhile, the time histories of sliding displacement and traction force in the stretching process were recorded to make comparison with the voltage signals measured by a voltmeter with the load resistance in the electrical circuit of 11 MΩ. In the mechanical test, a trapezoidal wave excitation was utilized with a frequency of 0.5 Hz and the displacement amplitude from 2.5 to 30 mm. Figure 4b shows the time histories of output voltage by the red line and the corresponding time histories of sliding displacement with amplitude of 30 mm and traction force by the green and blue lines, respectively. At the stage I, while the displacement between the tribo-pair increases with the traction force of the machine, the positive pulse of the output voltage is captured. And at the stage II, the output voltage shows opposite signals while the traction force is gradually canceled and the displacement decreases. The periodic characteristic of the voltage signals match well with those of the sliding displacement and traction force of the setting mechanical excitation, which demonstrates the feasibility of the TENG sensor for real-time breath monitoring. Furtherly, the voltage signals obtained varies obviously under different sliding amplitudes from 2.5 to 30 mm (Fig. 4c), which allows to investigate the effect of the displacement amplitude (i.e., the breathing depth). The variation tendency of the peak voltage versus the displacement amplitude is depicted in Fig. 4d. Obviously, the peak voltage increases linearly with the displacement amplitude and the variation relationship can be described as follows:

3 $$ {V}_{\mathrm{peak}}=0.01383{X}_{\mathrm{max}}+0.0092 $$

where *V*_peak_ is the peak value of the output voltage and the *X*_max_ means the maximum sliding displacement of the tribo-pair. The regulation in Eq. (3) reveals the relationship between the peak voltage and displacement excitation of the device with the applicable range “2.5 mm≤ *X*_max_ ≤30 mm”, which provides a basis for us to learn the effect of the abdominal circumference on the peak voltage and an accordance in predicting the peak voltage of the sensor in the breathing process. On the other hand, Figure 4d also illustrates that the useful electrical signals of the sensor can be captured with the amplitude of the traction force and the sliding displacement as small as 3.09 N and 2.5 mm, which means that the device can be easily driven by the variation of the abdominal circumference without causing uncomfortable feelings of the user.

### Respiration Monitoring

**Fig. 5:**
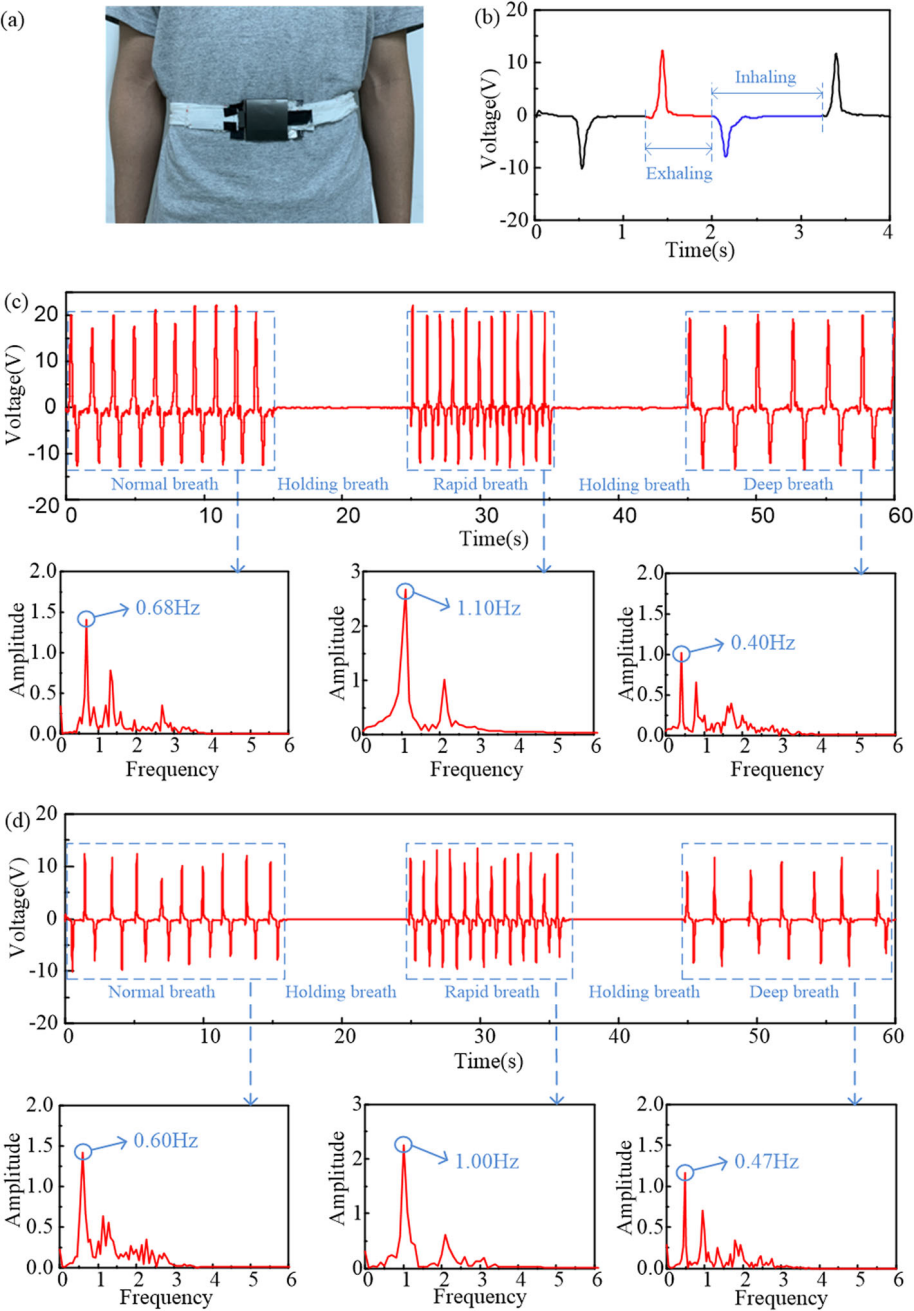
The sliding mode TENG respiration sensor for monitoring different breathing rhythms. **a** Photograph of the TENG sensor worn on the waist for respiration monitoring. **b** The correspondence between the output voltage signals and the processes of exhaling and inhaling in one breathing cycle. **c**, **d** Time histories of the output voltage signals for two volunteers with different waistlines (72.8 cm for **c** and 98.6 cm for **d**) and corresponding results of FFT for different breathing rhythms

To verify the feasibility of the device working as a respiration sensor, a set of real-time monitoring tests were carried out (Fig. 5a), and the electrical signals were measured via a voltmeter with the load resistance in the electrical circuit of 100 MΩ. During the breathing process, the belt of the device is kept in conformal contact with the user’s waist, and the variation of the user’s abdominal circumstance is reflected by the periodic reciprocating sliding oscillation of the tribo-pair. With the volunteer exhaling and inhaling periodically, the output voltage signals including pulses with positive and negative amplitudes appear. In actual applications, the captured electrical signals may contain more information related to the breathing process, i.e., the respiratory rates and the inhalation or exhalation process, etc. By illustrating the correlation between the periodic variation of voltage signals and the working mechanism of the respiration sensor, it will be more accurate to extract detailed information of breathing from the measured signals. Thus, we take one breathing cycle from the real-time tests as an example to illustrate the correlation (Fig. 5b). When a force is applied in the exhalation process, the tribo-pair slides outward and generates a pulse of the output voltage with positive amplitude as the accordance of the detection for exhalation process. Then correspondingly, when the applied force is revoked gradually in the inhalation process, the tribo-pair slides inward and generates a pulse of output voltage with negative amplitude as the accordance of the detection for inhalation process. Based on the abovementioned analyses, the voltage signals can be utilized to provide a deep understanding on breathing processes.

Furtherly, two volunteers, one aged 22 years old with a waistline of 72.8 cm and another aged 24 years old with a waistline of 98.6 cm, were invited to test the ability of the smart belt in reflecting specific breath behaviors of different individuals. To test the sensitivity of the device to different respiratory rates, the breathing processes offered by the volunteers involve three different breathing rhythms, i.e., normal, rapid, and deep breaths. During the breathing process with different rhythms, the electrical signals generated by the TENG sensor are successfully detected and shown in Fig 5c and d for the two volunteers, respectively. The voltage signals are repeatable and reliable for each rhythm, that presents obvious difference of respiratory rates in the breathing process. The time histories of the output voltage (Fig. 5c and d) for the two volunteers respectively exhibit steady variation (constant frequency and peak-valley value) in the processes of three breathing rhythms. Reflected by the results of fast Fourier transform (FFT) in Fig. 5c and d, the extracted frequency of the normal, rapid, and deep breaths are 0.68, 1.10, and 0.40 Hz, respectively for the 22-year-old volunteer and 0.60, 1.40, and 0.47 Hz for the 24-year-old one; those are reasonable respiratory rates for healthy adults [37]. It means that the key information of the respiratory rates can be collected via the electrical signals. On the other hand, the two volunteers in the tests are asked to hold breath to simulate the breathing pause caused by the symptom of apnea. Correspondingly, it is presented in Fig. 5c and d that the signals with value of zero volt last for about 10 s between two different breathing rhythms. It may be utilized as a judgment basis for OSAS and a further accordance for its diagnosis and warning. These results demonstrate that this TENG sensor can detect not only the respiratory rates but also the symptoms of the apnea.

**Fig. 6:**
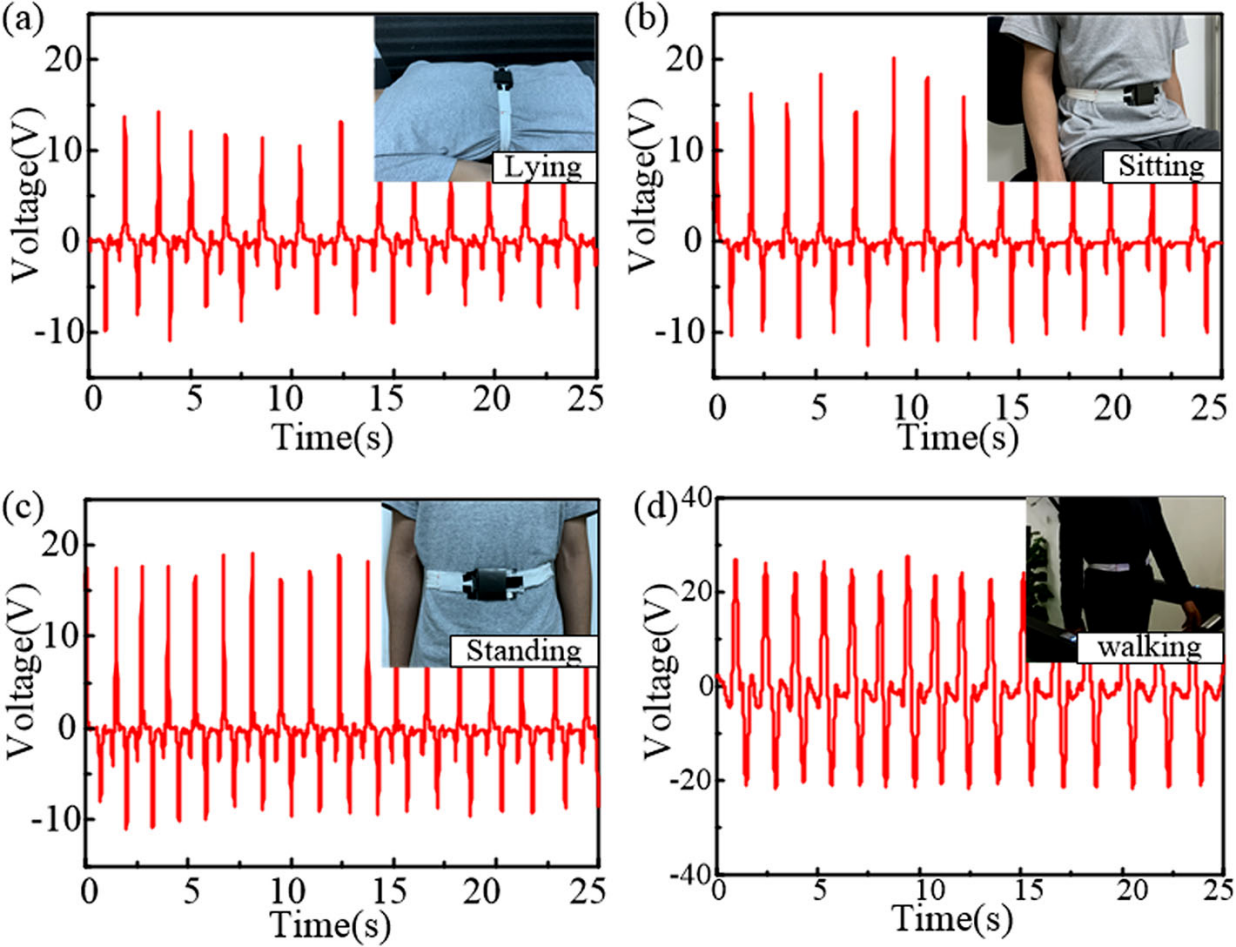
The TENG sensor for real-time respiration monitoring in different daily activities. The captured voltage signals and the corresponding testing photographs in processes of respiration monitoring when volunteer is (**a**) lying, (**b**) sitting, (**c**) standing, and (**d**) walking at a speed of 3 km/h

Moreover, a series of real-time tests were carried out by the volunteer in different states to confirm the practicability of the device in different daily activities. The voltage signals were measured via a voltmeter with load resistance of 100 MΩ in three different states, i.e., lying (case I in Fig. 6a), sitting (case II in Fig. 6b), standing (case III in Fig. 6c), and walking at a speed of 3 km/h (case IV in Fig. 6d). Figure 6a exhibits the obtained voltage signals with the volunteer lying to simulate the respiratory state during sleeping, while Fig. 6b-d present the captured voltage signals with the volunteer sitting, standing, and walking, respectively, to simulate the breathing processes in daytime activities. All the signals from cases I–IV show stable and continue voltage pulses in pace with the variation of the abdominal circumference during breathing, which coincide with the real processes of inhalation and exhalation. And the respiratory rates are respectively detected to be 0.54 Hz for case I, 0.52 Hz for case II, 0.72 Hz for case III, and 0.65 Hz for case IV. It is worth noting that there are some jitters existing in the signal waveform while walking in Fig. 6d, but the functionality for monitoring breathing rhythm is still achieved. The tests in the four cases demonstrate the feasibility of the respiration sensor as a wearable device for real-time respiration monitoring in different activities in daily life. Furtherly, we carried out a long-time continuous respiration monitoring for 180 s and the detected signals are presented in Additional file 1: Figure S1. The time histories of the output voltage exhibit stable alteration with the breathing processes during the tests, which demonstrate the stability of the TENG sensor for long-time monitoring in practical applications.

**Fig. 7:**
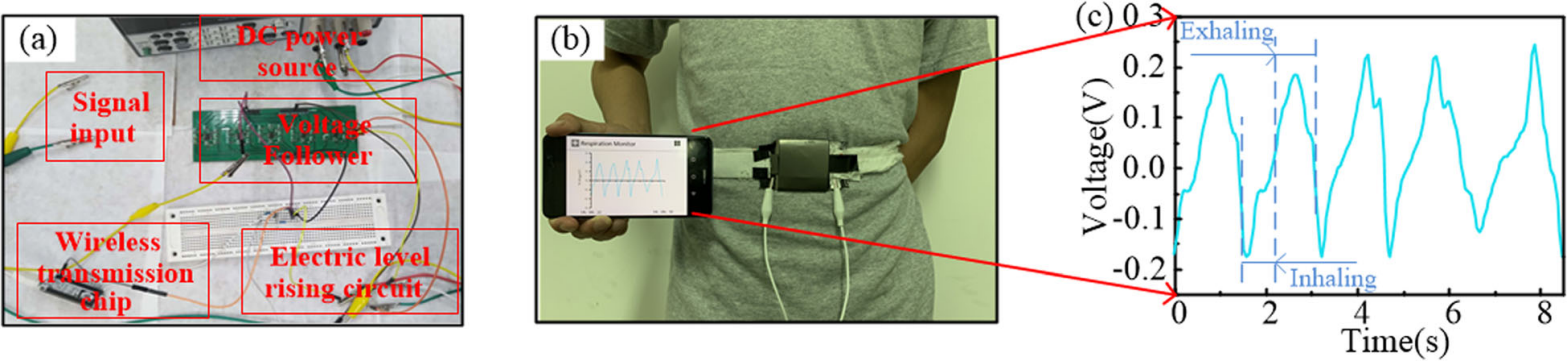
Real-time respiration monitoring via the TENG sensor with the wireless transmission system. **a** Photograph of the actual setup of the wireless transmission system. **b** Photograph of volunteer’s real-time breathing signals displayed on a mobile phone. **c** The respiratory waveform depicted with the data stored by the wireless transmission system

To further improve the portability of the device as a wearable respiration sensor, a wireless transmission system was designed for the exhibition of the breathing information on a mobile electronic equipment. Specifically, a real-time monitoring test equipped with the wireless transmission system proposed in Fig. 1b was carried out and the electrical signals generated by the TENG sensor were wirelessly transmitted and displayed on a cell phone. Figure 7a shows the actual setup of the wireless transmission system and Fig. 7b shows the signal waveforms containing breathing information displayed on the phone via the wireless transmission system. The measured respiratory information of the volunteer in Fig. 7b have been further processed on a PC and shown in Fig. 7c for better viewing. The depicted waveforms in Fig. 7c suggest that the respiratory rate is about 0.625 Hz. And the exhalation and the inhalation stages of the breathing process are identified and marked in Fig. 7c, which indicates the perfect reflection of the electric signals displayed on the phone to the actual respiratory status and the reliability and practicality of the wireless transmission system. To further demonstrate the accuracy of the wireless signals, voltmeter signals (with electrical load resistance of 10 MΩ) after TENG and wireless signals after wireless system were captured in the same breathing test and compared in Additional file 1: Figure S2. It is worth to be mentioned that the amplitude of the wireless signals is not the true value of the output voltage of the TENG sensor, but being processed proportionally. On the one hand, the signal width of the wireless signals is much wider than the voltmeter signals, which can be attributed to a comprehensive outcome of the larger input impedance of voltage follower (100 TΩ) in the wireless transmission chip, the existing load loss of the circuit and the low sampling rate which make the signals distorted slightly. On the other hand, though the waveform and the peak value are changed after the wireless system, the information about the breathing cycle delivered by the wireless signals coincides well with that of the voltmeter signals, which means that the respiratory rates can be correctly reflected by the signals obtained from the wireless transmission chip.

## Conclusions

In summary, we have designed and fabricated a waist-wearable wireless respiration sensor to monitor real-time respiratory status of humans in daily life and to transmit the breathing information to a mobile cell via a wireless transmission system. We furtherly illustrated its working mechanism in detail that it senses the variation of the abdominal circumference while breathing and output electrical signals containing rhythm information of the respiratory processes. In this study, theoretical analyses were performed to predict the output signals of the TENG and validate the possibility of the TENG to work as a respiration sensor. It was also demonstrated by a mechanical test that the sensor can be easily driven by a sliding displacement with an amplitude of 2.5 mm, which makes it feasible for use as a wearable sensor. To validate the applicability in reality, we carried out a series of tests by two volunteers to investigate the feasibility, accuracy, and sensitivity of the device to different individuals, different breathing rhythms, and different active states. The device was demonstrated applicable for not only the detection of apnea symptom but also the real-time monitoring of breath. Lastly, the wireless transmission system of the sensor was also proved to be efficient in wireless electrical signal transmission. Results stated above have shown the potential of the proposed sensor as a smart wearable respiration sensor and the household healthcare monitoring system comprehensively.

## Supplementary information

**Additional file 1:** **Figure S1.** A long-time continuous respiration monitoring for 180 s. Figure S2. Comparison of the voltmeter signals after TENG and wireless signals after wireless system which were captured in a same breathing test.

## Data Availability

The data and materials used are included in the manuscript.

## Abbreviations

ADC: Analog digital converter
FFT: Fast Fourier transform
OSAS: Obstructive sleep apnea syndrome
PTFE: Polytetrafluoroethylene
TENG: Triboelectric nanogenerator
