# Supplementary material for: A Portable Triboelectric Nanogenerator for Real-Time Respiration Monitoring
Source: Nanoscale Res Lett. 2019 Nov 28;14:354. doi: 10.1186/s11671-019-3187-4 (PMC6882997; doi:10.1186/s11671-019-3187-4)
Supplement: Supplementary file 1 — Additional file 1: Figure S1. A long-time continuous respiration monitoring for 180 s. Figure S2. Comparison of the voltmeter signals after TENG and wireless signals after wireless system which were captured in a same breathing test. [file 11671_2019_3187_MOESM1_ESM.docx]

**Supplementary Materials**

Figure. S1 A long-time continuous respiration monitoring for 180s

Figure. S2. Comparison of the voltmeter signals after TENG and wireless signals after wireless system that were captured in a same breathing test.
